# Impact and Sustainability of Foreign Medical Aid: A Qualitative Study with Honduran Healthcare Providers

**DOI:** 10.5334/aogh.3995

**Published:** 2023-03-03

**Authors:** Kara L. Faktor, Denise D. Payán, Alejandro J. Ramirez, Folasade P. May

**Affiliations:** 1 David Geffen School of Medicine at UCLA, Los Angeles, CA, 90095, USA; 2 Department of Health, Society, and Behavior, University of California, Irvine, Irvine, CA, 92697, USA; 3 Global Brigades, Ofibodegas Paseo Alto Local #2, Colonia Lara, Boulevard Los Proceres, Tegucigalpa, Honduras; 4 Vatche and Tamar Manoukian Division of Digestive Diseases, David Geffen School of Medicine at UCLA, Los Angeles, CA, 90095, USA; 5 UCLA Kaiser Permanente Center for Health Equity, Los Angeles, CA, 90095, USA

**Keywords:** foreign medical aid, short-term medical mission, healthcare providers, Honduras, impact, sustainability

## Abstract

**Background:**

There is growing concern about the sustainability and long-term impact of short-term medical missions (STMMs)—an increasingly common form of foreign medical aid—given that brief engagements do little to address the underlying poverty and fragmented healthcare system that plagues many low- and middle-income countries (LMICs). In the absence of formal evaluations, unintended but serious consequences for patients and local communities may arise, including a lack of continuity of patient care, poor alignment with community needs, and cultural and language barriers.

**Objective:**

We conducted semi-structured interviews with Honduran healthcare providers (n = 88) in 2015 to explore local providers’ perceptions of the impact and sustainability of foreign medical aid on patient needs, community health, and the country’s healthcare system.

**Methods:**

Respondents represented a random sample of Honduran healthcare providers (physicians, dentists, nurses) who worked for either a government-run rural clinic or non-governmental organization (NGO) in Honduras.

**Findings:**

Honduran healthcare providers largely framed foreign medical teams as being assets that help to advance community health through the provision of medical personnel and supplies. Nonetheless, most respondents identified strategies to improve implementation of STMMs and reduce negative impacts. Many respondents emphasized a need for culturally- and linguistically-tailored medical care and health education interventions. Participants also recommended strengthening local partnerships to mitigate the risk of dependence, including on-going training and support of community health workers to promote sustainable change.

**Conclusions:**

Guidelines informed by local Honduran expertise are needed to increase accountability for more robust training of foreign physicians in the provision of context-appropriate care. These findings provide valuable local perspectives from Honduran healthcare providers to improve the development and implementation of STMMs, informing strategies that can complement and strengthen healthcare systems in LMICs.

## Introduction

Short-term medical missions (STMMs) are a healthcare delivery model where services are provided for a limited period (days, weeks) to a community with high levels of unmet healthcare needs. The model is commonly employed in low- and middle-income countries (LMICs) by foreign providers and trainees from high-income countries (HICs). STMM team composition varies widely, ranging from university-affiliated to faith-based, and may include trainees in addition to healthcare providers. Physicians who participate in STMMs are often motivated by altruism and volunteer for a sense of renewal, patient gratitude, learning experiences, and legacy of teaching [1]. Some accounts suggest the benefit to the volunteer may equal or exceed that of the recipient, with time and resource costs to host communities [2,3]. Given the billions in yearly expenditure on STMMs, there is a lack of empirical data on long-term impacts and sustainability in LMICs [4,5].

Increasing concern about the sustainability of STMMs includes critiques about the brevity of these engagements to address the historical poverty and lack of healthcare infrastructure at the root of unmet healthcare needs in many LMICs [6,7,8]. Substantial concerns also exist as to whether HIC healthcare workers can provide contextually-appropriate medical care in these settings [4,9]. STMMs can contribute to healthcare system inefficiencies and quality issues, stemming from a lack of care continuity, poor alignment with community needs, and cultural and language barriers [10,11,12,13]. Questions have been raised about the impact on local healthcare providers, from undue burden to missed employment and training opportunities [12].

Assessing the impact of STMMs requires understanding the experience of local providers and patients [14,15]. Gathering data on the perspectives of local LMIC healthcare providers can facilitate development and implementation of effective non-governmental organization (NGO) health programming more closely aligned with local needs [16]. Challenges for NGO-sponsored STMMs include collaboration with local public health officials and contribution to infrastructure (e.g., water and sanitation projects) or health education programs [17]. While much of the literature focuses on the perspectives of foreign volunteers, recent studies have been conducted from the perspective of healthcare providers or patients from countries that receive foreign medical aid, given the consensus around the vital importance of these host country perspectives. [7,18,27,19,20,21,22,23,24,25,26]. This study examines the impact and sustainability of foreign medical aid from the perspective of Honduran healthcare providers and explores how well foreign medical aid is aligned with the health needs of Honduran communities. It uniquely incorporates both the perspectives of Honduran physicians, nurses, and dentists working directly for an international NGO, as well as those working for government-run clinics with which that NGO interfaces. We also provide policy and programmatic recommendations to improve the provision of medical aid by NGOs in resource-limited settings.

## Methods

### Setting

Honduras is ranked as a low middle-income country [28] where 17.9% of the population lives on less than $1.25 USD per day [29]. In 2018, health expenditures comprised 7% of the country’s GDP—around $146 USD was spent on health per capita [30]. The country’s healthcare system includes a public sector regulated by the Honduras Ministry of Health (SS) and a private sector, formed by for-profit and non-profit organizations. As part of the public sector, the Honduran Social Security Institute (IHSS) manages contributions from employers and employees [28]. Around 88.3% of the country’s population is covered by the SS—9% are registered with the IHSS and 2.7% are privately insured. Honduras has 3.9 physicians per 10,000 individuals—well below the 25 physicians per 10,000 population considered adequate by the World Health Organization (WHO) [29]. Most rural residents access local health centers. Smaller rural health centers (CESAR) are staffed by a nurse, while medical and dental health centers (CESAMO) in larger towns have a physician and nurses [28]. CESAR facilities are occasionally staffed by a physician in the social service year following medical school graduation. At many CESAR facilities, community health workers (CHWs) assist nurses with patient education at the facilities, as well community outreach and monitoring. These unpaid volunteers often receive training from either the government or an NGO. Patients requiring specialized care are referred to one of 30 regional or national hospitals that are concentrated in major urban areas, requiring significant travel time and costs for patients in remote areas. Moreover, based on the literature, Honduras has historically been one of the most frequent host countries for medical missions, especially those from the United States [31].

### Sampling and data collection

Respondents included government-employed physicians and nurses, as well as physicians and dentists employed by an American-led international health NGO. The NGO includes teams in six countries and works in South and Central Honduras to organize projects to meet the health and financial needs of Honduran communities through a student volunteer-empowered platform, employing both full-time and temporary Honduran staff [32]. The lead author was a volunteer for the NGO from 2014–2015.

In January 2015, a random number generator was used to select a sample from a registry of government-employed Honduran nurses, physicians, and dentists maintained by the NGO for collaboration purposes. Current employment by the government-run clinics of the staff on the registry was verified by telephone call to the clinics prior to randomization. A second random sample of physicians from a list of the NGO’s medical staff was selected using the same procedure. Invitations to participate were sent to 60% of providers from each list based on the randomly generated numbers.

The lead author developed a semi-structured interview script in consultation with NGO staff, which included questions on medical and public health needs, government-NGO collaboration, community health education, and preparedness of foreign providers. The script was piloted with Honduran nurses and physicians at three health centers (not included in the study) before being finalized.

Interviews were conducted in-person by the lead author in private offices in rural health centers (duration: 7–25 minutes). Interviews were nearly all in Spanish, and audio recorded with the participant’s written informed consent. Ethical approval for the study’s procedures and analysis was provided by UCLA’s Institutional Review Board (IRB#17-000379). The NGO did not have a local IRB process or Honduran academic affiliate at the time of data collection.

### Data analysis

Audio recordings were transcribed verbatim in the language in which the interviews were conducted (86 Spanish; 2 English per Honduran provider preference). The interviewer reviewed all transcripts to ensure accuracy. Transcript files were uploaded into a qualitative data analysis software, NVivo version 11 [33].

A combined inductive and deductive approach was used to analyze transcript content. Two investigators developed a preliminary codebook with key themes and sub-codes identified *a priori*, based on the research questions, instrument questions, field notes, and literature search [4,7,34,35,36,37,38]. An iterative process was undertaken where two investigators coded a random sub-set of transcripts (15%) to test the pilot codebook and identify emergent themes. After discussing results with a third investigator, the codebook was finalized to include 10 key themes (collaboration; community assets; community health needs; education and learning; foreign providers’ awareness, knowledge, skills, and capacities; organizational and local healthcare context; patient healthcare access barriers; perceived benefits; perceived disadvantages or harms; recommendations) and 49 codes. Under supervision, a research assistant used the final codebook to independently code all transcripts. Analytical summaries were iteratively reviewed and revised by the research team. All presented findings were stated by at least three respondents, with many reflecting five to 10 similar comments. Illustrative quotes were selected based on the most prevalent findings and translated into English.

## Results

Eighty-eight Honduran healthcare providers completed an interview (Table 1). Seventy-three percent worked for a government-run clinic (CESAR, CESAMO) and 27% worked for the NGO. A majority (65%) of physicians at government-run clinics had under six years of experience, with most having worked less than two years (38%). In comparison, around half (53%) of government-employed nurses had worked at their clinic six or more years, with some having served for decades in the community in which they lived.

**Table 1:** Honduran healthcare provider respondent professional experience & location, 2015.


	GOVERNMENT-EMPLOYED		NGO-EMPLOYED		
		
	PHYSICIAN* n = 26 n (%)	NURSE n = 38 n (%)	PHYSICIAN n = 13 n (%)	DENTIST n = 11 n (%)	TOTAL n = 88 n (%)

**Healthcare setting**					

CESAMO	26 (100)	22 (58)	–	–	48 (55)

CESAR	–	16 (42)	–	–	16 (18)

NGO STMM	–	–	13 (100)	11 (100)	24 (27)

**Organizational experience (years)**					

< 2 years	10 (38)	6 (16)	6 (46)	2 (18)	24 (27)

2–3 years	4 (15)	3 (8)	3 (23)	2 (18)	12 (14)

4–5 years	3 (12)	3 (8)	1 (8)	–	7 (8)

6–7 years	2 (8)	2 (5)	–	–	4 (5)

8 or more years	2 (8)	18 (47)	–	–	20 (23)

Unknown	5 (19)	6 (16)	3 (23)	7 (64)	21 (24)

**Estimated number of patients per day per provider (n = 64)**					

1–20 patients	6 (23)	8 (21)			14 (22)

21–40 patients	12 (46)	21 (55)			33 (52)

41+ patients	4 (15)	9 (24)			13 (20)

Unknown	4 (15)	–			4 (6)

**Clinic location (Department, n = 64)**					

Francisco Morazán	11 (42)	13 (34)			24 (38)

El Paraíso	13 (50)	23 (60)			36 (56)

Choluteca	1 (4)	1 (3)			2 (3)

La Paz	1 (4)	1 (3)			2 (3)


* Includes physicians in their social service year at CESAMOs and CESARs.

Government-employed participants had varying levels of experience with NGOs, faith-based organizations and universities who provided foreign medical aid. Familiarity with NGO-led STMMs ranged from direct participation in STMM organizational efforts to communication with NGOs between STMMs to provision of long-term care to patients who also received care through an NGO-led STMM. All respondents reported at least some familiarity with Honduran healthcare system-NGO collaborations.

### Local healthcare context

#### Community health needs

When asked about the needs of local Honduran communities, respondents reported a chronic shortage of local healthcare personnel, infrastructure, and medications. Most wished existing health centers had additional doctors and dentists. Government nurses described how having an insufficient number of providers limited medical care outside of business hours and impacted outreach activities, requiring prioritization of communities with the most severe disease burden. When asked about pressing health needs, diabetes and hypertension were most frequently mentioned, followed by diarrheal, respiratory and vector-borne illnesses. A nurse stressed that continuity of treatment for chronic illnesses was especially challenging, often requiring frequent medication changes due to shortages. Several health center nurses resorted to asking local politicians (e.g., mayors) to purchase medications when government supplies were insufficient.

#### Patient healthcare access barriers

Lack of healthcare access was felt to be particularly dire for patients in remote areas, including transportation challenges in accessing facilities: “It is difficult for patients, my patients have to walk three, four hours to obtain healthcare services” [CESAMO Physician]. Another significant barrier was out-of-pocket costs for medication or specialty care. Some government providers expressed frustration over the inability to provide appropriate treatment post-diagnosis due to a lack of available medications or a distant pharmacy. Most respondents reiterated how poverty prevented patients from purchasing medication. A government nurse explained how lack of medication access drove attendance at NGO-led STMMs: “I think the majority attend due to a lack of money to pay for medication they might need in the future if they were to get sick” [NGO Physician]. Providers also explained how socioeconomic status frequently precluded patients from paying for follow-up care or specialist referrals. Financial constraints were compounded by shortages in ambulances, fuel, personnel, and other health center resources for outreach to the most distant communities—resulting in an increased reliance on CHWs.

#### Community assets

Given these challenges in accessing medical care, health education and prevention were emphasized by participants as being essential. Many highlighted the need for hygiene education to prevent diarrheal and vector-borne illnesses: “The majority of diseases come from not taking care of hygiene—personal hygiene, hygiene at home, and a lack of toilets. Most diseases that are here are preventable” [CESAMO Physician]. Dental providers emphasized the need to increase awareness of oral hygiene, including use of a toothbrush by children. Many nurses reported providing health education in collaboration with CHWs, educating patients about boiling and treating water, eliminating standing water outdoors, ventilating smoke from indoor stoves, and adequate nutrition. Health education sessions were often held during vaccination campaigns or community sanitation events.

### Perceived STMM benefits

Honduran providers mentioned several benefits of foreign medical aid in addressing healthcare system inadequacies (Table 2). A primary contribution was the provision of medical personnel and supplies, including medications that were unavailable or too expensive. Several respondents referenced patients whose prescriptions went unfilled without foreign aid, emphasizing a diagnosis was meaningless without access to treatment. Many nurses reported submitting medication requests to the study NGO to compensate for shortages in government-supplied medications. As one nurse explained, such donations enabled health centers to ensure more consistent access to medications between STMMs.

**Table 2:** Perceived benefits and disadvantages of Short-term Medical Missions (STMMs) and recommendations for improvement based on interviews with Honduran healthcare providers.


	PERCEIVED BENEFITS	PERCEIVED DISADVANTAGES	RECOMMENDATIONS

*Medications & Supplies*	Increased access to medications during the STMMs, often at no cost to patients Avenues for health center providers to request medication donations from the NGO between STMMs to supplement government supply	Patient reporting of false symptoms or attending multiple STMM days to obtain medications for later use (unnecessary prescriptions) Inconsistent access to the same medication for chronic conditions	Formation of country-specific medication guidelines with reference to the WHO essential medicines list

*Foreign Medical Providers*	Increased access to physician consultations, including specialist care Perceptions of exceptional empathy for the patient	Expectation of specialist physicians providing primary care Limited knowledge of the local culture and socioeconomic context Devaluing of local physicians	Education of foreign providers regarding local ethics-informed practices Pre-STMM meetings between local and foreign providers

*Provision of Care*	Extension of primary care services to more remote areas Increased access to specialized services (e.g., dental, gynecology)	Lack of coordination with local providers for follow-up at government health centers in-between STMMs Undue burden on local providers to follow-up on test results from STMM consultations Inadequate prioritization of health needs Language barriers with additional need for qualified interpreters	Strengthening of partnerships with local providers to guide provision of care during STMMs Inclusion of community leaders in planning efforts

*Health Education & Prevention*	Increased access to screening services (e.g., Pap smear) Provision of antiparasitic medications	Language and cultural barriers limiting the effectiveness of education sessions	Training opportunities and resources for CHWs Visual aids for patient education sessions to address low literacy levels


Respondents also reported that foreign medical providers supplemented local public health education and prevention efforts. One provider remarked:

They provide education which allows the patient to have the curative and preventative part. For me, the preventative part is best. A brigade came and provided deworming medication, but they also told [people] to wash their hands…and motivated this [behavioral] change among patients [CESAR Nurse].

Several respondents said foreign providers appeared to be more empathetic, trustworthy, and understanding compared to local providers. One Honduran provider remarked,

They listen to the patient. If the patient begins to cry, they give them a moment to cry. Meanwhile, Honduran doctors are less sensitive because we have experienced so much here that we lose our sensitivity…I have seen how they have more heart [NGO Dentist].

Many attributed elevated trust and confidence in foreigners to high-quality education and advanced technology available in HICs.

### Perceived disadvantages of STMMs

Challenges associated with STMMs included unnecessary prescriptions, lack of coordination with local providers, and inadequate prioritization of health needs (Table 2). Some community members accessed healthcare through STMMs more frequently than at government health centers. A respondent credited this difference to the perception that every patient received medication during STMMs. According to several respondents, patients frequently reported false symptoms or attended multiple mobile clinics to stockpile medications in case of future illness. One physician stated, “…there is a patient that isn’t sick when he arrives, but he comes with a list of medications” [CESAMO Physician]. While health center providers felt most patients appropriately received medications and used them as intended, some nurses provided examples of patients who expressed confusion about medications from a STMM, while others emphasized the issue of excess use of antibiotics.

Several respondents expressed concern about lack of local clinic-based follow-up care and coordination with local providers. When follow-up occurred, there was a resultant burden on the local healthcare system since many patients were not already receiving care at a health center. The need to link NGO and routine healthcare system services—and to expand geographic reach—was said to be essential to promote care continuity and to create national impact.

Apprehension about foreign providers’ transition to a resource-limited environment was expressed. Providers described how foreign physicians demonstrated more caution during consultations, which was attributed to lack of familiarity with local illnesses or over-reliance on laboratory diagnostics. Several respondents conveyed the challenges for foreign dentists to adapt to a mobile clinic setting without specialized equipment. Foreign dentists were perceived to be more willing to extract teeth without full consideration of a patient’s nutritional status. Given the large number of patients seen in a STMM, limited time meant focusing solely on the most urgent issue. Others noted that the large quantity of foreign specialists diminished focus on primary care.

### Gaps in training for context-appropriate care by foreign providers

Although foreign providers were perceived by some to be more knowledgeable due to training in a well-resourced setting, language barriers contributed to a loss of information or misunderstanding during physician-patient exchanges. Need for interpreters with medical terminology training was identified as being critically important. With an appropriate interpreter, most respondents felt the language barrier didn’t considerably affect consultation quality. Simple greetings in Spanish by foreign providers were said to increase patients’ trust in non-Spanish-speaking physicians.

A need for knowledge about the healthcare system, cultural norms, and socioeconomic conditions was emphasized. Respondents expressed that foreign providers should be better prepared to work in resource-limited settings: “They have to prepare and know the politics, economy, history, and diseases. That is the bare minimum training [that] doctors need…I think this type of preparation is needed for any professional that is visiting a new country” [CESAMO Physician].

Local providers felt foreign physicians needed more orientation to local pathologies and available medications, emphasizing preventive medicine. Some highlighted the need for a holistic understanding of the healthcare system to situate patients’ requests in a local context. Some dentists described consultations where a patient’s meager financial circumstances were not considered:

We need to understand the physical problems of patients. For example, you should ask the patient if they are diabetic or hypertensive, if they ate this morning and if they took their medication before you anesthetize them and perform extractions…If you extract too many teeth from a patient, and if the patient lives on a mountain…the patient may die [NGO Dentist].

### Recommendations for improved impact and sustainability

#### Strengthening collaboration with local partners

Respondents acknowledged the importance of local collaboration, including coordinating with local authorities (e.g., mayor, faith leader) to prepare for upcoming STMMs and outreach activities. Participants recommended NGOs allocate resources and personnel to increase local collaborative partnerships, saying that: “People like the mayors, for example, have a larger role and more influence over the people” [CESAMO Physician]. One respondent stated that planning meetings were valuable to facilitate direction. A local NGO physician reinforced this notion, conveying that teamwork between NGO providers and local community volunteers was necessary to achieve an organized STMM.

Respondents recommended Honduran and foreign healthcare providers meet prior to a STMM to share contextual knowledge. Meetings were viewed as an opportunity for foreign physicians to clarify doubts, solicit guidance about medication lists and discuss when patient referrals were appropriate. Thus, respondents advocated for additional preparatory meetings with integration of dental and medical services.

Outside of STMMs, respondents described the need to collaborate to provide health supplies and education. For health center providers, routine communication between the NGO and local partners about medication requests was seen as vital to address community needs in-between STMMs. Some respondents voiced concerns about the absence of a link between the patient and the nearest health center when care was provided in a STMM setting. Indiscriminate use of antibiotics and medication non-adherence, especially among patients with asymptomatic hypertension, were concerns of government and NGO providers alike. Several government providers suggested increasing coordination of care between Honduran NGO-employed providers and health center staff to ensure appropriate follow-up for patients with chronic conditions or those awaiting test results.

#### Investments in community health education

Several respondents detailed the need for the NGO to support an increase in training resources, including educational materials, supplies and transportation costs for CHWs to compensate for limited outreach capacity, particularly in rural communities in-between STMMs. Multiple providers viewed CHWs as a vital link between nurses and community members—connecting pregnant women and children to preventive care and providing routine community surveillance for emerging health issues. CHWs serve a crucial role in disseminating health messages, with one nurse explaining how information was spread in informal networks: “[CHWs] promote [healthy behaviors] in their families and cause a multiplicative effect because they tell a neighbor, comment to a friend, and, little by little, they inform everyone” [CESAR Physician].

As community members, CHWs were perceived as having greater credibility. Government nurses described CHWs as the “eyes and ears in the communities” and as “pillars” supporting the clinics [CESAR Nurse]. In communities with active CHWs, respondents perceived lower rates of diarrheal and respiratory illnesses following use of latrines and eco-stoves, as well as a perceived decrease in infant and maternal mortality due to prenatal care.

Reiterating their importance, several respondents emphasized the need to provide continuous capacity building and training opportunities for unpaid CHWs. These respondents acknowledged that in practice, CHWs need motivation: “We have to keep motivating them, giving them lectures, and educating them so they can be prepared for the community. If we don’t continue this momentum, they become less motivated” [CESAR Nurse].

Several nurses felt CHWs should receive more education on diarrheal and respiratory illness, chronic diseases, family planning, and hygiene practices. Community-initiated sanitation campaigns, hand-washing stations in schools, and routine use of latrines were said to have lessened the burden of diarrheal illnesses. Others stressed that CHWs should be able to provide first aid and vaccinations. One NGO physician recommended further training of CHWs to lead educational sessions during the STMMs, noting they often led sessions without grasping the “weight of the information” [NGO Physician].

Individual patient education was emphasized as a means for sustained impact, with some physicians indicating it was the provider’s responsibility during consultations. Most CESAR nurses reported having a schedule of daily or weekly sessions for patients, often enlisting CHW assistance. Topics were frequently based on pressing community health concerns, including group sessions for hypertension, diabetes and prenatal care.

Many acknowledged challenges associated with language barriers and low patient literacy, stressing the need to tailor sessions to local dialects. As one respondent explained: “The advantage of a community member giving the lecture is that it’s communicated through the native language of the community and the community is able to understand the message” [NGO Physician]. Others recommended using more visual aids in educational sessions, and one nurse underscored the importance of timely repetition to promote information retention. Several providers insisted that health education should be focused on younger populations to combat taboos and myths: “The youth are the foundation because it’s harder to bring awareness to others, they already have their customs and traditions” [CESAMO Nurse].

## Discussion

### Strengthening of local partnerships

Examining perceptions of local providers in LMICs on the impact and sustainability of care provided by foreign physicians during STMMs is crucial given the widespread use of the model. Cultivation of dependence on foreign medical aid, especially when care is delivered in a manner that is siloed from routine health services, is among the most common critiques in the literature, as STMM efforts may disincentivize government investment in healthcare infrastructure [4,7,9,10,11,37]. In this study, respondents identified lack of medication access as a key driver of STMM attendance. Community members were said to routinely seek out care through STMMs instead of government health centers, potentially undercutting demand for local healthcare services. While STMMs aim to address medication needs, isolation from local healthcare infrastructure risks dependence [39]. As respondents noted, patients receiving care during a STMM were often not connected to government health centers, making follow-up for chronic conditions challenging. Furthermore, follow-up for services rendered during a STMM (e.g., Pap smear results) could place an undue burden on an already fractured healthcare system, as discussed in other studies [12]. Involvement of local providers to help guide STMM care delivery and to strengthen accountability of the government healthcare system has been recommended, thereby mitigating the disempowerment of local communities [12,40].

Likewise, health center staff in our study advocated for increased coordination of care to facilitate follow-up, especially for chronic conditions [41]. Open communication about medication requests between health center staff and NGOs was viewed as fundamental to bridge the gap between patient medication needs and government supply in-between STMMs, though risks releasing the government from accountability for consistent supply of essential medications. STMM coordination must also extend beyond health center staff to strengthening relationships with local community leaders. Respondents expressed the importance of pre-STMM planning meetings to facilitate direction and give a voice to community leaders and volunteers, minimizing the potential burden on recipient communities and improving quality of care.

### Positive perceptions of caring and belief in inherent benefit

In this study, STMM participants were framed by several respondents as being exceptionally empathetic, as well as inspiring elevated patient confidence in part due to notions of more advanced training in HICs. In reality, foreign physicians often provide care without accountability to the local healthcare system and without long-term physician-patient relationships, which may facilitate short-term focused attention and enhanced expressions of empathy. Patient discourse representing foreign physicians as superior to local colleagues can be harmful, perpetuating power imbalances and reinforcing neo-colonialism [22,42]. Devaluing local assets and skills is a detriment for all involved given skill and knowledge transfer should be bidirectional [39,43]. Beliefs in the inherent benefits of STMMs make evaluation of the impact on health outcomes difficult to ascertain [39].

### Investing in health education

In a systematic review of STMMs, about half (48%) included an educational element [4]. Some view the exchange as a mechanism of bidirectional skill and knowledge transfer between local and foreign providers [44]. Other educational components of STMMs focus on patient health education. Respondents highlighted the challenges of low literacy, advising use of additional visual aids and active engagement of participants, which has been shown to be effective in similar settings [45,46]. The NGO supporting the STMMs described in this study provided some training for CHWs, who were viewed by respondents as having credibility in encouraging modification of health-related behaviors. Health center and NGO providers alike emphasized the importance of on-going training and motivation to address high attrition, which has been noted elsewhere when relying on unpaid CHWs [47]. Sufficient compensation, healthcare system support, and well-defined job responsibilities are critical factors for sustainable implementation of CHW programs [48,49]. Further investigation into the educational components of STMMs is needed, both to ascertain the effectiveness of knowledge transfer among providers and the impact on community health literacy.

### Context-driven ethics

Although there is broad consensus on promoting ethically-delivered medical care, application of a context-appropriate ethical framework remains a challenge for many STMMs [50]. Advanced disease and malnutrition can decrease the efficacy of certain interventions or make them altogether dangerous for particular patients in resource-limited settings. For this reason, HIC providers must consider a patient’s complex medical condition to avoid inappropriate or harmful intervention [9]. A Honduran dentist in our study described one such situation where a patient’s nutritional status and distance to the nearest health center needed to be factored into a decision about whether to extract multiple teeth. While procedural interventions magnify the need for context-driven ethical guidelines, safe and effective prescribing practices also require insight into local ethics-informed practices [51]. Honduran providers highlighted the challenge of providing care for chronic conditions after a STMM, stressing frequent changes in medication due to shortages and formulary differences. These challenges underscore the need for in-country pharmacists to ensure that STMMs adhere to the WHO essential medicines list and country guidelines, and a coordinated local-external approach to ensure appropriate follow-up [51].

Respondents in this study elaborated on tendencies for patients to report false symptoms to obtain medications for potential future illness. Ready distribution of medications can lead to patient confusion over intended medication use, which was reported (albeit infrequently) by nurses [52]. While prescribing was similar between local and foreign physicians in a Dominican Republic study, there were discrepancies in prescribing practices, especially for antibiotics and anti-parasitic medications [51]. Foreign clinicians can be perceived as being excessively reliant on diagnostic tests—revealing lack of insight into local healthcare infrastructure [51]. Thus, efforts should be aimed at robust preparation of foreign physicians for STMMs. Some discrepancies in care delivery between local and foreign providers can be mitigated through regular evaluation of patient outcomes and establishment of quality metrics using embedded research in care delivery processes for strengthened accountability [39,51,53]. Although health impact assessment tools exist, little has been done to validate such tools or encourage their adoption [35].

### Adequate preparation of foreign providers

Many published guidelines include recommendations for provider preparation prior to a STMM [39]. Understanding health system dynamics and patient perspectives requires knowledge of a country’s social, political, and economic conditions [12], as well as local pathologies and health needs. Interviews with local providers elsewhere have emphasized the need for preparation with respect to language, culture, and a country’s conditions [54]. Surveys of organizations supporting STMMs indicate inadequate volunteer preparation to understand the local context, thus undervaluing local knowledge [12,23]. Sufficient preparation of foreign physicians to understand the structural determinants of health in a host country requires insight into socio-political and public health conditions, as well as self-awareness of one’s personal culture-driven attitudes that inform care delivery [42,52]. Honduran physicians advocated for pre-STMM meetings between local and foreign providers to enable visiting physicians to learn about referral processes and to solicit guidance about medication formularies, especially for specialists. Such orientation can alleviate burden on local staff, strengthen integration with local healthcare infrastructure, and improve quality of care [10,51]. While historically many meetings have been conducted in-person after arrival in-country, remote collaboration is feasible to facilitate robust preparation and meaningful partnership engagement, as demonstrated by STMM activities during the COVID-19 pandemic. The acceptability and impact of remote preparation among foreign physicians and subsequent effects on patient outcomes is a topic for further inquiry.

### Strengths and limitations

Our study has several strengths including random selection as the sampling method for this qualitative study, which increases the likelihood that the results are generalizable to the population of Honduran providers involved with STMMs. The study also incorporates respondents from peri-urban and remote areas, including different provider types. Limitations include a lack of data on patient perspectives for comparison. Further, providers’ responses were based on service-focused interactions with predominantly secular NGOs and do not reflect education-focused university partnerships or other subsets of global health partnerships.

## Conclusions

While Honduran healthcare providers largely framed foreign medical teams in positive terms, most identified avenues to improve this increasingly common model of external assistance. Recommendations included strengthening local partnerships to mitigate risk of dependence, including on-going training and support of CHWs to promote sustainable change. Clinical guidelines reflecting local expertise and practice are needed to increase accountability for more robust training of foreign physicians in the provision of context-appropriate and linguistically-tailored care [55]. Findings have programmatic implications for organizations working to develop and implement STMMs that effectively complement resources available within a complex, yet evolving, healthcare system in a culturally-appropriate and sustainable manner.

## Data Accessibility Statement

Expanded excerpts can be provided for specific themes on reasonable request.
